# Non-DHFR-mediated effects of methotrexate in osteosarcoma cell lines: epigenetic alterations and enhanced cell differentiation

**DOI:** 10.1186/s12935-016-0289-2

**Published:** 2016-02-29

**Authors:** Martin Sramek, Jakub Neradil, Jaroslav Sterba, Renata Veselska

**Affiliations:** Laboratory of Tumor Biology, Department of Experimental Biology, Faculty of Science, Masaryk University, Kotlarska 2, 611 37 Brno, Czech Republic; Department of Pediatric Oncology, University Hospital Brno and Faculty of Medicine, Masaryk University, Cernopolni 9, 613 00 Brno, Czech Republic

**Keywords:** Methotrexate, Osteosarcoma, Epigenetic regulation, DNA methylation, Histone acetylation, All-*trans* retinoic acid, Osteogenic differentiation

## Abstract

**Background:**

Methotrexate is an important chemotherapeutic drug widely known as an inhibitor of dihydrofolate reductase (DHFR) which inhibits the reduction of folic acid. DHFR-mediated effects are apparently responsible for its primary antineoplastic action. However, other non-DHFR-mediated effects of methotrexate have been recently discovered, which might be very useful in the development of new strategies for the treatment of pediatric malignancies. The principal goal of this study was to analyze the possible impact of clinically achievable methotrexate levels on cell proliferation, mechanisms of epigenetic regulation (DNA methylation and histone acetylation), induced differentiation and the expression of differentiation-related genes in six osteosarcoma cell lines.

**Methods:**

The Saos-2 reference cell line and five other patient-derived osteosarcoma cell lines were chosen for this study. The MTT assay was used to assess cell proliferation, DNA methylation and histone acetylation were detected using ELISA, and western blotting was used for a detailed analysis of histone acetylation. The expression of differentiation-related genes was quantified using RT-qPCR and the course of cell differentiation was evaluated using Alizarin Red S staining, which detects the level of extracellular matrix mineralization.

**Results:**

Methotrexate significantly decreased the proliferation of Saos-2 cells exclusively, suggesting that this reference cell line was sensitive to the DHFR-mediated effects of methotrexate. In contrast, other results indicated non-DHFR-mediated effects in patient-derived cell lines. Methotrexate-induced DNA demethylation was detected in almost all of them; methotrexate was able to lower the level of 5-methylcytosine in treated cells, and this effect was similar to the effect of 5-aza-2′-deoxycytidine. Furthermore, methotrexate increased the level of acetylated histone H3 in the OSA-06 cell line. Methotrexate also enhanced all-*trans* retinoic acid-induced cell differentiation in three patient-derived osteosarcoma cell lines, and the modulation of expression of the differentiation-related genes was also shown.

**Conclusions:**

Overall non-DHFR-mediated effects of methotrexate were detected in the patient-derived osteosarcoma cell lines. Methotrexate acts as an epigenetic modifier and has a potential impact on cell differentiation and the expression of related genes. Furthermore, the combination of methotrexate and all-*trans* retinoic acid can be effective as a differentiation therapy for osteosarcoma.

## Background

Methotrexate (MTX; amethopterin; 4-amino-10-methylfolic acid), a structural analogue of folic acid, is a chemotherapeutic drug which is still very frequently used as a treatment of osteosarcomas—the most common primary malignant bone tumors affecting both children and adults [1]. MTX has been included in therapeutic protocols for many years, but its dosage and administration schedules are still being optimized [2, 3].

MTX enters the cell through an active transport mechanism and by facilitated diffusion, and once inside, it is converted into polyglutamate MTX by folylpolyglutamyl synthase [4–6]. Polyglutamate MTX reversibly inhibits dihydrofolate reductase (DHFR) but also inhibits other enzymes, for example, phosphoribosylaminoimidazolecarboxamide formyltransferase (AICAR transformylase) or thymidylate synthase (TS). Inhibition of DHFR affects the reduction of folic acid and consequently leads to a lack of 5,10-methylenetetrahydrofolate, which is used as a coenzyme in the biosynthesis of thymidine. Moreover, TS is directly blocked by MTX and by unmetabolized dihydrofolate. Purine precursor biosynthesis is also affected by the deficiency of another folate co-factor, 10-formyltetrahydrofolate and by MTX inhibition of AICAR transformylase. The inhibition of dTMP and purine synthesis causes MTX-induced cell death [7].

Although MTX is able to inhibit proliferation and/or induce apoptosis in neoplastic cells, there is also evidence that it induces differentiation. MTX was able to induce differentiation in colon cancer cells primarily due to the intracellular depletion of purines [8], in immature and undifferentiated monocytic cells [9] and in rat choriocarcinoma cells [10]. Overall, cytostatic, cytotoxic and differentiation effects are mediated by the functional suppression of DHFR and nucleotide biosynthesis.

In addition to the cytostatic and differentiation effects of MTX, non-DHFR-mediated effects concerning the modulation of important epigenetics determinants have also been described, such as DNA methylation [11] and histone acetylation [12]. The mechanism of the methylation of biomolecules is not always clear because both the DHFR- and non-DHFR-mediated effects of MTX can contribute to the decreased methylation of molecules in the cell. On one hand, inhibition of folate metabolism as described above can affect the intracellular levels of 5-methyltetrahydrofolate which transfers methyl groups to methionine synthase to generate methionine from homocysteine [13]. Methionine can be utilized for the synthesis of the universal methyl donor S-adenosylmethionine (SAM) which plays a pivotal role in the generation of 5-methylcytosine. On the other hand, MTX directly inhibits methionine adenosyltransferase (MAT) mRNA expression and reduces MAT protein levels which significantly decreases MAT activity [13]. This is of particular importance because MAT is a key enzyme that catalyzes the only reaction that produces SAM. Moreover, MAT expression and activity can be inhibited even by a very low concentration of MTX (50 nmol). Regarding histone acetylation, molecular modeling suggested that MTX is a potential histone deacetylase inhibitor due to its shared structural similarity with some histone deacetylase inhibitors (e.g., butyrate or trichostatin A), and it has been shown that MTX directly inhibits histone deacetylase activity and induces histone H3 acetylation in vitro [12].

It has been shown that the induced differentiation of tumor cells is a promising strategy in cancer therapy [14]. Especially, all-*trans* retinoic acid (ATRA) and its derivatives are widely used differentiation drugs that can induce the osteogenic differentiation of osteosarcoma cells [15]. The main disadvantage of retinoid usage is the occurrence of resistance [16]. On one hand, DNA methylation has a significant role in preventing normal differentiation in pediatric cancers [17], and on the other hand, DNA demethylation can contribute to cell differentiation; for example, the expression of the retinoic acid receptor beta (*RARB*) can be activated by the hypomethylating action of 5-aza-2′-deoxycytidine [18]. Histones are involved in the regulation of chromatin structure and gene expression as well as in DNA methylation. Histone H3 acetylation is also associated with gene expression. Therefore, due to its impact on nucleotide synthesis, as well as DNA methylation and histone acetylation, MTX could modulate gene expression and enhance the ATRA-induced differentiation of osteosarcoma cells.

In the present study, we focused on MTX action in six cell lines derived from osteosarcomas. The MTX effect on DNA methylation was compared with the effect of the known DNA methyltransferase inhibitor 5-aza-2′-deoxycytidine (5AZA), and the accumulation of acetyl histone H3 after MTX treatment was compared with the effects of the known histone deacetylase inhibitors sodium butyrate (BUT) and sodium valproate (VAL). We also studied the MTX impact on the expression of selected genes related to cell differentiation, and we assessed cell differentiation induced by MTX, ATRA or a combination of the two. Therefore, our work represents the first complex study of the non-DHFR-mediated effects of MTX in cancer cells with special attention to the modulation of epigenetic information in terms of DNA methylation and histone acetylation.

## Results

Our results showed that the non-DHFR mediated effects of MTX were detectable, especially in patient-derived cell lines and that MTX act as an epigenetic modifier with an impact both on DNA demethylation and the accumulation of acetylated histones. Moreover, the combination of MTX and ATRA may represent a new therapeutic option in the differentiation therapy of osteosarcoma.

### Patient-derived cell lines are more resistant to MTX than Saos-2 cell line

**Fig. 1:**
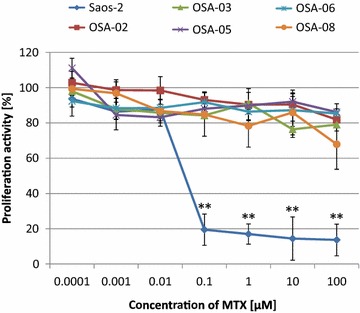
Proliferation activity of osteosarcoma cell lines after treatment with MTX. Proliferation activity was measured using MTT assay at day 6 of incubation with various concentrations of MTX and compared with those of untreated control cells. Untreated controls were set as 100 %. The data represent the mean ± SD. Experiments were repeated three times in duplicates. **P < 0.01, indicates significant differences from the respective cell lines

Using the MTT assay, an analysis of proliferation activity was performed on day 6 of MTX treatment at concentrations from 0.0001 to 100 μM. Significant differences in the sensitivity of cell lines used in this study (Fig. 1) were noted. On one hand, MTX showed a strong cytotoxic effect on the Saos-2 reference cell line at concentrations ranging from 0.1 to 100 μM. On the other hand, all five patient-derived OSA cell lines were significantly more resistant to MTX action, and even the very high concentration of 100 μM was not sufficient to reach the IC_50_.

### MTX induces DNA demethylation in a majority of osteosarcoma cell lines

**Fig. 2:**
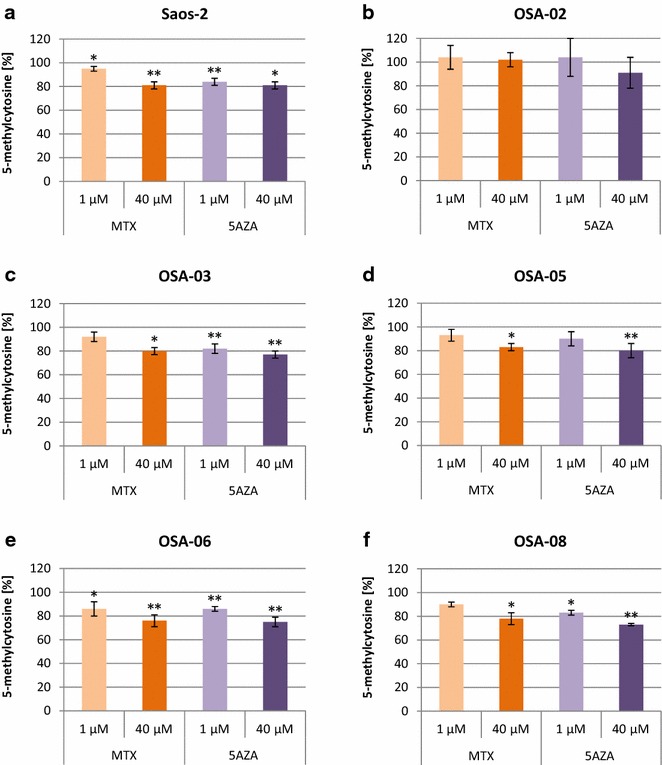
Changes in DNA methylation in osteosarcoma cell lines after treatment with MTX. Levels of 5-methylcytosine in Saos-2 (**a**), OSA-02 (**b**), OSA-03 (**c**), OSA-05 (**d**), OSA-06 (**e**) and OSA-08 (**f**) cells as measured using an ELISA assay at day 3 of incubation. The levels of 5-methylcytosine are presented as a percentage change compared to the levels found in untreated control cells. Untreated controls were set as 100 %. The data represent the mean ± SD. Experiments were repeated three times. *P < 0.05, **P < 0.01, indicate significant differences from the respective control groups

Despite the mild effect of MTX on cell proliferation, we continued to study the non-DHFR-mediated effects of MTX on DNA methylation. Significant DNA demethylation was observed in Saos-2, OSA-03, OSA-05, OSA-06 and OSA-08 cells at day 3 of the MTX treatment, especially at a concentration of 40 μM (Fig. 2). The MTX-induced DNA demethylation was most obvious in the OSA-06 cells–the level of 5-methylcytosine decreased to 86 % at 1 μM MTX and to 76 % at 40 μM MTX in comparison with untreated control cells. As expected, the positive control 5AZA induced DNA demethylation in Saos-2, OSA-03, OSA-05, OSA-06 and OSA-08. Surprisingly in Saos-2, OSA-03, OSA-05 and OSA-06 cells, 40 μM MTX induced DNA demethylation comparable to the effect of 5AZA at the same concentration. We did not observe any changes in DNA methylation in OSA-02 cells.

### MTX increases the global histone H3 acetylation in OSA-06 cells

**Fig. 3:**
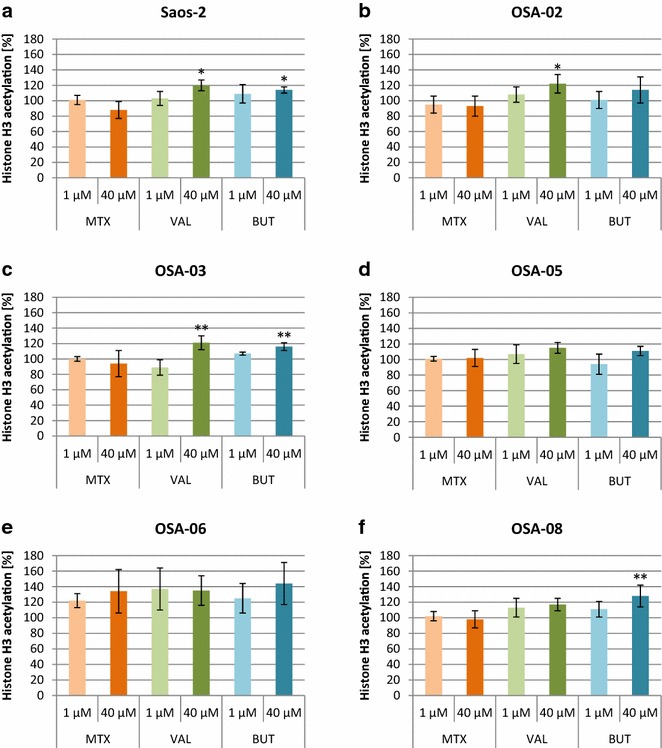
Changes in global histone H3 acetylation in osteosarcoma cell lines after treatment with MTX. Levels of global histone H3 acetylation in Saos-2 (**a**), OSA-02 (**b**), OSA-03 (**c**), OSA-05 (**d**), OSA-06 (**e**) and OSA-08 (**f**) cells as measured using ELISA assay at day 3 of incubation. The levels of global histone H3 acetylation are presented as a percentage change compared to the levels found in untreated control cells. Untreated controls were set as 100 %. The data represent the mean ± SD. Experiments were repeated three times. *P < 0.05, **P < 0.01, indicate significant differences from the respective control groups

**Fig. 4:**
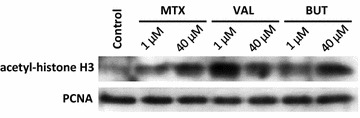
Effect of MTX on histone H3 acetylation in OSA-06 cells. Cells were incubated with 1 or 40 μM MTX, VAL or BUT. At day 3, nuclear protein extracts were harvested and immunoblotted by anti-acetyl-histone H3 antibody. PCNA served as a loading control

Given that MTX is a possible histone deacetylase inhibitor, we determined whether MTX could increase histone H3 acetylation. Treatment with BUT and VAL served as a positive controls and, in some cases, treated cells showed the significant accumulation of acetylated histone H3 in comparison with an untreated control (Fig. 3). We did not detect an increase in the global acetylation of histone H3 at day 3 of the MTX treatment in Saos-2, OSA-02, OSA-03, OSA-05 and OSA-08 cells (Fig. 3a–d, f); however, we observed an increase of histone H3 acetylation in OSA-06 cells (Fig. 3e), and therefore, this cell line was further analyzed using western blotting. Cells were incubated with MTX, VAL or BUT, and the nuclear protein fractions were harvested and immunoblotted on day 3 of the treatment (Fig. 4). Our data demonstrated that MTX increased histone H3 acetylation in OSA-06 cells in a concentration-dependent manner.

### MTX alters the expression of differentiation-related genes

**Fig. 5:**
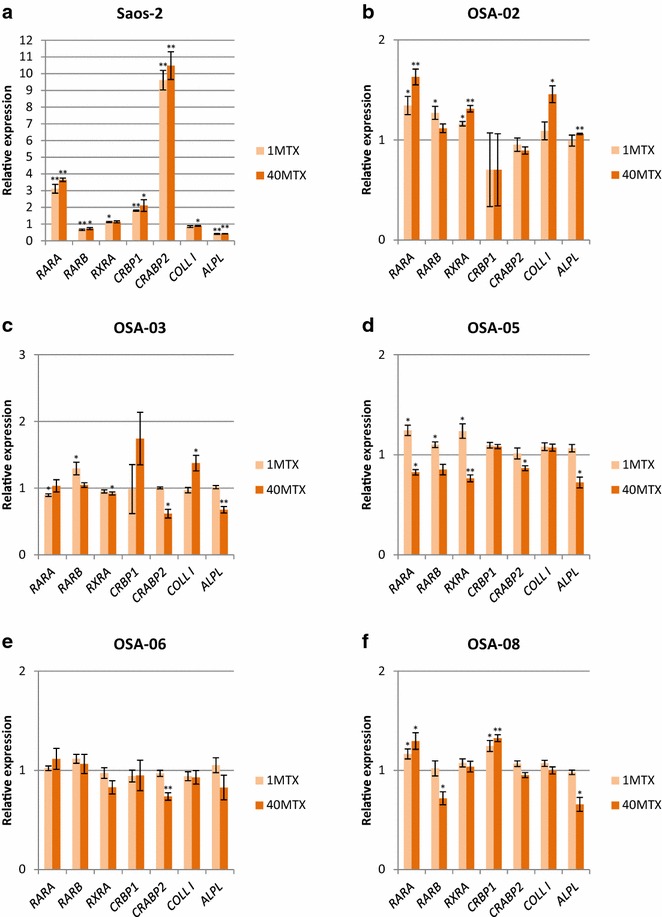
Changes in expression of differentiation-related genes in osteosarcoma cell lines after treatment with MTX. The relative expression of selected genes in Saos-2 (**a**), OSA-02 (**b**), OSA-03 (**c**), OSA-05 (**d**), OSA-06 (**e**) and OSA-08 (**f**) cells measured using RT-qPCR at day 3 of incubation with 1 μM MTX (1MTX) or with 40 μM MTX (40MTX). The levels of relative gene expression are presented as fold changes compared to the levels detected in control samples. The data represent the mean ± SD. Experiments were repeated three times. *P < 0.05, **P < 0.01, indicate significant differences from the respective control groups

To further explore the importance of epigenetic alterations induced by MTX, we decided to assess the MTX impact on the expression of selected genes involved in cell differentiation. The expression of genes encoding known markers of osteogenic differentiation (*COLLI*, *ALPL*) as well as genes involved in ATRA metabolism and the regulation of gene expression were evaluated using RT-qPCR on day 3 of MTX treatment at concentrations of 1 μM and 40 μM (Fig. 5). In Saos-2 cells, we observed a significant increase in the expression of *RARA*, *CRBP1* and *CRABP2*. Interestingly, the expression of *CRABP2* was increased approximately ten-fold, but the expression of *RARB* and *ALPL* was significantly lower (Fig. 5a). In OSA-02 cells, MTX at both concentrations significantly increased the expression of *RARA* and also the expression of *COLLI* at 40 µM. In contrast, the expression of *CRBP1* was at a very low level (Fig. 5b). In OSA-03 cells, *COLLI* expression was significantly higher after treatment with 40 µM MTX, but the same concentration of MTX significantly decreased the expression of *CRABP2* and *ALPL* (Fig. 5c). In OSA-05 cells, 1 µM MTX significantly increased the expression of *RARA*, *RARB* and *RXRA* (Fig. 5d). In OSA-06 cells, MTX significantly decreased the expression of *CRABP2* only (Fig. 5e). In OSA-08 cells, the expression of *RARA* and *CRBP1* was significantly increased after MTX treatment (Fig. 5f).

### Osteogenic differentiation is enhanced by combined treatment with MTX and ATRA

**Fig. 6:**
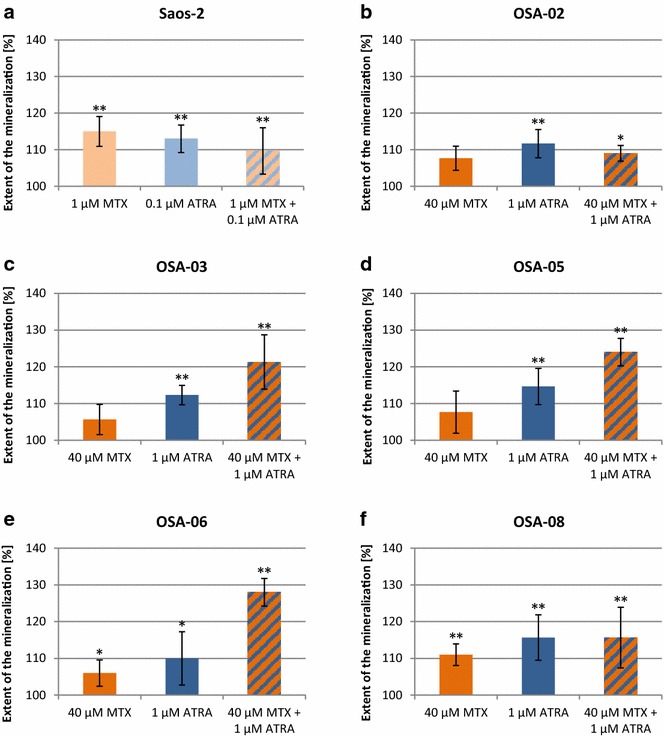
Changes in matrix mineralization in osteosarcoma cell lines after treatment with MTX. Saos-2 (**a**), OSA-02 (**b**), OSA-03 (**c**), OSA-05 (**d**), OSA-06 (**e**) and OSA-08 (**f**) cell lines treated with MTX and/or ATRA were measured using staining with Alizarin Red S at day 21 of incubation. The extent of mineralization is presented as a percentage change compared to the levels found in untreated control cells. Untreated controls were set as 100 %. The data represent the mean ± SD. Experiments were repeated three times. *P < 0.05, **P < 0.01, indicate significant differences from the respective control groups

As indicated by the previous analyses, MTX treatment significantly increased the expression of some genes involved in ATRA metabolism and the regulation of gene expression. This observation led us to explore whether MTX could enhance ATRA-induced differentiation. After 21 days of cultivation, all cell lines formed calcium-positive nodules in control cell populations as well as under all experimental conditions. In the Saos-2 cell line, MTX significantly enhanced the extent of this mineralization (Fig. 6a), but MTX-induced mineralization was less apparent in all five OSA cell lines (Fig. 6b–f). ATRA significantly enhanced the mineralization in Saos-2 cells in a manner similar to MTX. In all OSA cell lines ATRA was always more effective in enhancing mineralization than MTX and in all cases significantly enhanced the extent of the mineralization. The combination of ATRA (0.1 or 1 μM) and MTX (1 or 40 μM) did not have an additional effect on the amount of calcium sediments in Saos-2, OSA-02 and OSA-08 cell lines. Interestingly, in the OSA-03, OSA-05 and OSA-06 cell lines, a combined treatment with ATRA and MTX significantly enhanced the mineralization in comparison with the untreated control by 21–28 %. The greatest increase in mineralization with the combined treatment was found in the OSA-06 cell line (Fig. 6e).

## Discussion

For decades, MTX was a commonly used therapy in osteosarcoma patients [19]. MTX interferes with folate metabolism but its other antineoplastic effects are still being discovered, and these effects can be helpful in the development of new strategies for osteosarcoma treatment [20]. The principal goals of this study were to analyze the non-DHFR-mediated effects of MTX in cell lines derived from osteosarcomas and to determine whether MTX acts as an epigenetic modifier in terms of DNA demethylation, histone acetylation, subsequent changes in gene expression and induced cell differentiation. The Saos-2 osteosarcoma cell line was chosen as the reference cell line for this study, and it was compared with five other cell lines that were derived in our laboratory from biopsy samples taken from patients suffering with osteosarcoma [21].

The MTT assay indicated that Saos-2 cells were very sensitive to MTX treatment, which showed a strong cytotoxic effect in these cells at 0.1 μM. This observation is in full accordance with our previous study [22] and with results obtained by other research groups studying the sensitivity of Saos-2 cells to MTX [23]. All five OSA cell lines, which were derived from diagnostic biopsies of primary tumors without any previous neoadjuvant chemotherapy, were significantly more resistant to the DHFR-mediated effect of MTX than Saos-2 cell line. The resistance of the OSA cell lines is surprising when we consider that 40 μM MTX is comparable with the peak of the MTX plasma concentration achieved during high dose-MTX treatments of pediatric hematological malignancies. In osteosarcomas, the peak MTX levels are approximately 1000 μM but rapidly decline within hours. Altogether, these results show that lower levels of MTX could not fully inhibit DHFR and nucleotide biosynthesis in all OSA cell lines despite prolonged exposure [24]. Furthermore, all OSA cells showed a low doubling time in comparison with Saos-2 cells that could diminish the proliferation-dependent cytotoxicity of MTX [25]. Other possible mechanisms of MTX resistance are an augmented drug efflux, impaired intracellular polyglutamation or alterations in the activity of target enzymes [6].

Because we did not observe any profound negative DHFR-mediated impact of MTX on cell proliferation in almost all of the cell lines included in this study, we continued with experiments that focused on other possible non-DHFR-mediated effects of MTX on osteosarcoma cells. MTX decreases the concentration of 5-methyltetrahydrofolate [26, 27] and reduces MAT expression and activity [13], which can further affect methylation in treated cells. 5-methyltetrahydrofolate and homocysteine are two important molecules in methionine biosynthesis [28]. Methionine reacts with ATP, and SAM is formed as a product. This key reaction is catalyzed by MAT. A methyl group from SAM is enzymatically transferred to the 5-position of cytosine to generate 5-methylcytosine in genomic DNA. Our data demonstrate that MTX significantly decreased 5-methylcytosine levels in genomic DNA and induce global genomic DNA demethylation. Surprisingly, significant DNA demethylation was observed in almost all of the cell lines used in our experiments.

Due to the similar structure of MTX and known histone deacetylase inhibitors, e.g., butyrate and trichostatin A, MTX can inhibit histone deacetylase activity and induce histone H3 acetylation [12]. Nevertheless, the five cell lines in our study including Saos-2 showed a poor response to MTX in this aspect. Only the OSA-06 cell line has a higher level of acetyl histone H3 after MTX treatment. Therefore, OSA-06 cells were further analyzed by western blotting to confirm this effect and the results showed that MTX increased the level of acetylated histone H3 in this cell line. As expected, most of cell lines showed a significant increase in the amount of acetylated histone H3 after treatment with BUT or VAL.

In contrast, MTX changed the methylation status of DNA in almost all of the studied cell lines. This finding led us to explore two important issues: (i) alterations of expression of the selected genes in MTX-treated cells and (ii) effect of MTX on differentiation in osteosarcoma cells after a combined treatment with ATRA because ATRA is a widely used inducer of differentiation in osteosarcoma cells [15, 29, 30].

Both of these aspects are important and mutually interconnected. Inducing differentiation of tumor cells by retinoids seems to be a very promising strategy, but it can be complicated by the resistance of tumor cells [16, 31–33]. The regulation of cell differentiation by retinoids is mediated by two types of nuclear receptors: retinoic acid receptors (RAR) and retinoid X receptors (RXR). DNA methylation patterns could affect the normal course of the expression of genes involved in cell differentiation [34]. For instance, *RARB* is methylated in many breast cancer cell lines and treatment of these cell lines with a demethylating agent can restore inducibility of *RARB* by ATRA [35]. Other studies have demonstrated that the *RARB* promoter is hypermethylated in colorectal and lung carcinomas and that this methylation could account for the *RARB* downregulation [18, 36].

At first, we studied whether MTX could modulate the expression of genes involved in retinoid/ATRA metabolism and signaling and whether MTX alone could induce differentiation in osteosarcoma cells. After treatment with MTX, we observed that the *RARA* was significantly highly expressed in Saos-2 and OSA-02 cells. Another interesting observation was the significantly increased expression of *CRABP2* in Saos-2 cells. *CRABP2* encodes the cytosol-to-nuclear shuttling protein, which facilitates the binding of retinoic acid to its receptor and the transfer of this complex to the nucleus. Furthermore, the expression *CRBP1*, which encodes the carrier protein involved in the transport of retinol from liver storage site to the peripheral tissue was also significantly elevated in Saos-2 cells as well as in OSA-08 cells.

Regarding osteogenic differentiation, we examined the expression of known osteogenic differentiation markers, i.e., collagen type I (*COLLI*) and alkaline phosphatase (*ALPL*) [30]. Increase in *COLLI* expression is typical in the early stages of differentiation whereas levels of *ALPL* usually increase during the process of mineralization, i.e., during the late stages of induced differentiation [37]. Nevertheless, we did not observe a marked increase in expression of these markers.

Because the expression of some differentiation-related genes was modulated after 3 days of MTX treatment, we decided to evaluate a long time course of osteogenic differentiation using mineralization measured by Alizarin Red S staining [30]. MTX and ATRA alone increased the extent of matrix mineralization in all cell lines but ATRA was apparently more effective. Interestingly, MTX alone was able to induce cell differentiation effectively in the Saos-2 cell line; this finding is in accordance with previously published results on choriocarcinoma cells [38]. Our data also demonstrated that a combined treatment with ATRA and MTX enhanced matrix mineralization most greatly in the OSA-03, OSA-05 and OSA-06 cell lines, so the combined administration of MTX and retinoids could be effective in differentiation therapy of some osteosarcomas.

## Conclusions

To summarize, our study represents the first complex analysis of the non-DHFR-mediated effects of MTX on cell lines derived from osteosarcomas. We showed that MTX treatment significantly decreased the proliferation activity in the Saos-2 reference cell line, but all five patient-derived OSA cell lines were much less sensitive to MTX action. These results suggest that all OSA cell lines were not sensitive to the DHFR-mediated effects of MTX at concentrations used. More importantly, our results provide the evidence for non-DHFR-mediated effects of MTX in both Saos-2 and OSA cell lines. MTX could act as an epigenetic modifier because (1) it induced significant DNA demethylation in almost all of the studied osteosarcoma cells and (2) it increased the global acetylation of histone H3 in OSA-06 cells. Our findings also demonstrated the modulation of the expression of differentiation-related genes by MTX at certain concentrations. The most important result of our study showed that ATRA-induced cell differentiation might be enhanced by the combined treatment of cells with MTX; this implies new possibilities in administration of these drugs in clinical practice.

## Methods

### Cell culture

The Saos-2 cell line (No. HTB-85) was purchased from the American Type Culture Collection (Manassas, VA, USA). The OSA-02, OSA-03, OSA-05, OSA-06 and OSA-08 cell lines were derived in our laboratory from tumor samples obtained from patients surgically treated for osteosarcoma as previously described [21]. A description of the cell lines included in this study and their responses to MTX is provided in Table 1. The Research Ethics Committee of the School of Medicine (Masaryk University, Brno, Czech Republic) approved the study protocol and a written statement of informed consent was obtained from each patient or his/her legal guardian.

**Table 1:** Description of the cell lines and characterization of their responses to MTX

Cell line	Gender	Age	Tumor type	Time of biopsy	DNA demethylation	Increased histone H3 acetylation	MTX + ATRA enhanced differentiation
Saos-2	F	11	N/A	N/A	Y	N	N
OSA-02	M	21	HGCC	DG	N	N	N
OSA-03	M	15	HGCC	DG	Y	N	Y
OSA-05	M	9	T	DG	Y	N	Y
OSA-06	F	16	O	DG	Y	Y	Y
OSA-08	M	10	O	DG	Y	N	N

Gender: *M* male, *F* female; Age at the time of diagnosis: years; Tumor type: *HGCC* high grade conventional-chondroblastic, *T* teleangiectatic, *O* osteoblastic, *N/A* information not available; Time of biopsy: *DG* diagnostic, *N/A* information not available; Responses to MTX, i.e. DNA demethylation, histone H3 acetylation; enhanced matrix mineralization: *Y* yes, *N* no

Cells were grown in Dulbecco’s modified Eagle’s medium (DMEM) supplemented with 10 % (Saos-2) or 20 % (OSA-02, OSA-03, OSA-05, OSA-06 and OSA-08) fetal bovine serum, 100 IU/ml penicillin, 100 mg/ml streptomycin, and 2 mM glutamine (all purchased from GE Healthcare Europe GmbH, Freiburg, Germany). Cell culture was performed under standard conditions at 37 °C in a humidified atmosphere containing 5 % CO_2_.

### Chemicals

MTX (Sigma-Aldrich, St. Louis, MO, USA) was prepared as a stock solution at a concentration of 20 mM in 1 M NaOH (Sigma) and stored at −20 °C under light-free conditions. BUT and VAL (both from Sigma) were prepared as stock solutions at concentrations of 50 mM in sterile PBS and 5AZA (Sigma) was prepared as a stock solution at a concentration of 1 mM in sterile PBS. All three stock solutions were prepared freshly for each use. ATRA (Sigma) was prepared as a stock solution at concentration of 100 mM in DMSO (Sigma) and stored at −20 °C under light-free conditions.

For the determination of proliferation activity, seven different concentrations of MTX ranging from 0.0001 to 100 μM were tested. For all other experiments, concentrations of 1 and 40 μM MTX were used. 5AZA, VAL and BUT served as positive controls and were used at the same concentration as MTX, i.e., 1 and 40 μM.

In experiments on matrix mineralization, 1 μM ATRA was used as in previously published experiments concerning the ATRA-induced differentiation of osteosarcoma cells [30]. For the treatment of Saos-2 cells lower concentrations (i.e., 1 μM MTX and 0.1 μM ATRA) were used due to the previously reported sensitivity of these cells [30].

### MTT assay

To evaluate cell proliferation, the MTT assay was used to detect the activity of mitochondrial dehydrogenases in living cells. 96-well plates were seeded with 1 × 10^4^ cells per well in 200 μl of culture medium, and the cells were allowed to adhere overnight. The medium was removed and fresh medium containing the selected concentrations of chemicals described above or a control medium was added. The plates were incubated under standard conditions. To evaluate changes in cell proliferation, the medium was removed and replaced with 200 μl of fresh DMEM containing 3-[4,5-dimethylthiazol-2-yl]-2,5-diphenyltetrazolium bromide (MTT) at 0.5 mg per ml. The plates were then incubated at 37 °C for 2.5 h. The medium was carefully removed, and the formazan crystals were dissolved in 200 μl of DMSO. The absorbance with a reference absorbance at 620 nm was measured at 570 nm using a Sunrise Absorbance Reader (Tecan, Männedorf, Switzerland).

### DNA methylation analysis

Total DNA was extracted using a DNeasy Blood & Tissue Kit (Qiagen, Hilden, Germany), and its concentration and purity was determined spectrophotometrically. Levels of 5-methylcytosine were detected using a 5-mC DNA ELISA Kit (Zymo Research Corporation, Irvine, CA, USA) according to the manufacturer’s instructions. The absorbance was measured at 450 nm with the Sunrise Absorbance Reader.

### Global histone H3 acetylation

For the specific measurement of global histone H3 acetylation, an EpiQuik Global Histone H3 Acetylation Assay Kit (Epigentek Group Inc., Farmingdale, NY, USA) was used according to the manufacturer’s instructions. The absorbance was measured at 450 nm using the Sunrise Absorbance Reader.

### RT-qPCR

The relative expression levels of selected genes were studied using RT-qPCR. Total RNA was extracted using the GenElute™ Mammalian Total RNA Miniprep kit (Sigma), and its concentration and integrity was determined spectrophotometrically. For all samples, equal amounts of RNA (i.e., 25 ng of RNA per 1 μl of total reaction volume) were reverse transcribed into cDNA using M-MLV (Top-Bio, Prague, Czech Republic). RT-qPCR was carried out in 10 μl using KAPA SYBR^®^ FAST qPCR Kit (Kapa Biosystems, Wilmington, MA, USA) and analyzed using 7500 Fast Real-Time PCR System and 7500 Software v. 2.0.6 (both Life Technologies, Carlsbad, CA, USA). Changes in the transcript levels were calculated using Cq values standardized to a housekeeping gene (*HSP90AB1*), used as an endogenous reference gene control. Primers used for retinoic acid receptor alpha (*RARA*), retinoic acid receptor beta (*RARB*), retinoid X receptor alpha (*RXRA*), retinol binding protein 1 (*RBP1*), cellular retinoic acid binding protein 2 (*CRABP2*), collagen type I (*COLL I*), alkaline phosphatase (*ALPL*) and heat shock protein (*HSP90AB1*) are described in Table 2.

**Table 2:** Sequences of the primers used for qPCR

Gene	Primer sequence	Product length (bp)
*RARA*	F: 5′-CGACCGAAACAAGAAGAAGAAGG-3′ R: 5′-TTCTGAGCTGTTGTTCGTAGTGT-3′	166
*RARB*	F: 5′-TGATGGAGTTGGGTGGACTT-3′ R: 5′-GCTTGGGACGAGTTCCTCAG-3′	288
*RXRA*	F: 5′-CTCAATGGCGTCCTCAAGGT-3′ R: 5′-CACTCCATAGTGCTTGCCTGA-3′	111
*RBP1*	F: 5′-TGACCGCAAGTGCATGACAA-3′ R: 5′-GACCACACCTTCCACTCTCA-3′	142
*CRABP2*	F: 5′-TGCTGAGGAAGATTGCTGTG-3′ R: 5′-CCCATTTCACCAGGCTCTTA-3′	183
*COLL I*	F: 5′-CAGACTGGCAACCTCAAGAA-3′ R: 5′-GGAGGTCTTGGTGGTTTTGT-3′	180
*ALPL*	F: 5′-CCACGTCTTCACATTTGGTG-3′ R: 5′-AGACTGCGCCTGGTAGTTGT-3′	196
*HSP90AB1*	F: 5′-CGCATGAAGGAGACACAGAA-3′ R: 5′-TCCCATCAAATTCCTTGAGC-3′	169

### Western blot analysis

Nuclear protein extracts were harvested using NE-PER™ Nuclear and Cytoplasmic Extraction Reagents (Life Technologies, Carlsbad, CA, USA) according to the manufacturer’s instructions. Total proteins (15 μg) were loaded onto 10 % polyacrylamide gels, electrophoresed, and blotted on polyvinylidene difluoride membrane (Bio-Rad Laboratories, Munich, Germany). The membranes were blocked with 5 % nonfat dry milk in PBS with 0.1 % Tween-20 (Sigma) and incubated overnight either with rabbit polyclonal anti-acetyl-Histone H3 (Ac-Lys^9^) (No. H9286, Sigma, dilution 1:1000) or with mouse monoclonal anti-Proliferating Cell Nuclear Antigen (anti-PCNA) (No. P8825, clone PC10, Sigma, dilution 1:3000). Antimouse IgG antibody peroxidase conjugate (No. A9917, Sigma, dilution 1:10,000) or anti-rabbit IgG HRP-linked antibody (No. 7074, Cell Signaling Technology, Danvers, MA, USA, dilution 1:2000) was used as the secondary antibodies. ECL-Plus detection was performed according to the manufacturer’s instructions (GE Healthcare, Little Chalfont, UK).

### Alizarin Red S staining

Levels of extracellular matrix mineralization were evaluated using Alizarin Red S staining, which detects calcium compounds both in tissue sections and in vitro. The cells were seeded onto 12-well plates at concentrations of 1 × 10^4^ (Saos-2 cell line) or 5 × 10^3^ (all OSA cell lines) cells per well and were cultivated in the presence or absence of ATRA and/or MTX for 21 days. The cultivation medium with these substances was renewed every 7 days. After 21 days of incubation, the medium was removed, the cells were washed with PBS and fixed with 3 % paraformaldehyde in PBS at room temperature for 20 min. Subsequently, the cells were incubated with 2 % Alizarin Red S (Sigma) at room temperature for 45 min. Thereafter, the cells were washed five times with deionized water and then with 70 % ethanol for 30 s. Red Alizarin dye was then dissolved via incubation with 100 mM cetylpyridinium chloride (Sigma) at 50 °C for 60 min. The absorbance was measured at 450 nm also using the Sunrise Absorbance Reader.

### Statistical analysis

The quantitative data are shown as mean ± SD of three independent experiments. Data from MTT assays were analyzed using two-way ANOVA followed by the Scheffé post hoc test. *P* < 0.01 was considered significant. The other data were analyzed using Student’s t test. *P* < 0.05 (two-sides) were considered statistically significant.

## Abbreviations

AICAR transformylase: phosphoribosylaminoimidazolecarboxamide formyltransferase
5AZA: 5-aza-2′-deoxycytidine
ATRA: all-*trans* retinoic acid
BUT: sodium butyrate
DHFR: dihydrofolate reductase
MAT: methionine adenosyltransferase
MTT: (3-(4,5-dimethylthiazol-2-yl)-2,5-diphenyltetrazolium bromide
MTX: methotrexate
PCNA: proliferating cell nuclear antigen
SAM: S-adenosylmethionine
TS: thymidylate synthase
VAL: sodium valproate
